# Reproducing the physiological environment of HIV infection

**Published:** 2013-09

**Figure:**
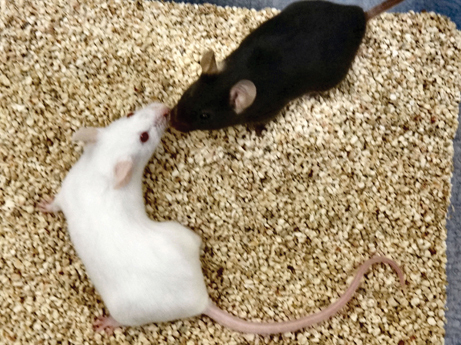


Acquired immunodeficiency syndrome (AIDS), caused by human immunodeficiency virus (HIV) infection, is characterised by increased vulnerability to potentially life-threatening infections. Although there are treatments that can impede progress of the disease, there are no cures or vaccines. The most common route for transmission of the virus is via heterosexual intercourse. The animal models that are currently available do not accurately replicate the physiological environment of vaginal intercourse, so the effects of factors such as seminal fluid composition on HIV infectivity have not been thoroughly examined. Here, Mary Jane Potash and colleagues describe a system for reproducible and efficient sexual transmission of HIV in mice. Using this model, the group show that the rate of viral transmission dramatically declines during estrus in mice, demonstrating that the local environment in the female reproductive tract can influence viral infectivity. The study provides an effective *in vivo* system for investigating the influence of physiological factors on HIV infection and for testing the therapeutic potential of new strategies for intervention. **Page 1292**

